# Dynamic changes in protist community composition along a surface water-groundwater transect in the Danube wetland Lobau, Vienna, Austria

**DOI:** 10.3389/fmicb.2026.1749803

**Published:** 2026-03-12

**Authors:** Angela Cukusic, Clemens Karwautz, Christina Murhammer, Grit Rasch, Marharyta Biletska, Christian Griebler

**Affiliations:** Department of Functional and Evolutionary Ecology, University of Vienna, Vienna, Austria

**Keywords:** groundwater ecology, microeukaryotes, groundwater protist communities, protist diversity, surface-groundwater environmental gradient

## Abstract

Groundwater remains an underexplored habitat for protistan diversity and community dynamics relative to better-studied surface aquatic environments. To address this knowledge gap, we compared protistan communities at two sites, a wetland surface water body and a nearby shallow aquifer, using molecular analysis, to shed light on environmental drivers of both total and active protist communities. The 2-year time series with monthly sampling provided insight into seasonal patterns, and barcoding enabled taxonomic assignment. Our study identified differences in community composition and trophic modes associated with habitat type. Protistan communities in shallow groundwater exhibited pronounced seasonal dynamics, apparently temporally linked to their surface water counterparts. Higher absolute water levels in the backwater than in groundwater, along with a significant fraction of phototrophic protists sampled from the shallow aquifer, are consistent with groundwater recharge from surface water influencing the groundwater protistan community composition.

## 1 Introduction

Investigating microbial communities in transition zones is crucial for understanding community assembly, adaptations to changing environmental conditions, ecosystem processes, and the diversity of life on Earth. The importance of surface-subsurface exchanges within the hydrological cycle for biogeochemical processes and biodiversity has been repeatedly emphasized (Malard et al., 2002; Hou et al., 2017). We also know that groundwater-dependent ecosystems, such as wetlands, perform critical watershed functions that shape spatio-temporal dynamics and influence the rates of material and energy fluxes (Lane et al., 2023; Ward et al., 1999). Water movement between surface waters and groundwater triggers and underpins changes in physicochemical conditions, actively transporting microorganisms from one location subject to specific selection pressures [selection, drift, speciation, and dispersion (Hanson et al., 2012)] to another. The transport of unicellular organisms between terrestrial soil and aquatic habitats appears to occur frequently (Sieber et al., 2020) via passive dispersal routes to the subsurface through groundwater recharge from seepage and surface waters.

Aquatic protists are a heterogeneous group of microeukaryotes characterized by complex phylogenetic relationships, diverse trophic modes, and a wide range of ecological roles. They contribute to key biogeochemical cycles and connect the microbial world with aquatic and terrestrial metazoan communities (Gadd and Raven, 2010; Azam et al., 1983). Compared with bacteria and archaea, protists have a more restricted range of metabolic capabilities encoded in their genomes. However, this range encompasses primary production and mixotrophy, as well as grazing by prokaryotic cells and virus particles, and a parasitic lifestyle (Sherr and Sherr, 1988; Dodds and Whiles, 2010; Falkowski et al., 2008). Their functional potential and niche space are better defined than those of prokaryotes. Nevertheless, ecological studies of protist communities often focus on just a few environments, such as marine environments or surface inland waters (Bik et al., 2012). Research on groundwater protists is scarce and partly outdated, largely based on laborious morphological identification (Sinclair and Ghiorse, 1987; Hirsch and Rades-Rohkohl, 1983), and quantitative data from microscopic counts (Novarino et al., 1997; Karwautz et al., 2022), as well as molecular analysis using fingerprinting methods (Euringer and Lueders, 2008). Most studies have focused either on contamination plumes or on ecosystems that are not representative of general groundwater properties (Kinner et al., 2002; Novarino et al., 1994; Holmes et al., 2013; Oliverio et al., 2018; Borgonie et al., 2015). However, notable exceptions that combine cultivation-based approaches with quantitative assessments and low-throughput sequencing have substantially advanced our understanding of groundwater protistan communities (Risse-Buhl et al., 2013; Loquay et al., 2009).

Although the use of new high-throughput sequencing technologies has recently emerged as an effective way to identify protist contributions to the groundwater food web (Herrmann et al., 2020), little progress has been made in linking distribution to driving environmental conditions. The use of 18S rDNA barcoding to link environmental and spatial drivers of protist communities has so far been focused primarily on soils (Oliverio et al., 2020; Huang et al., 2023), marine (Egge et al., 2021; Caracciolo et al., 2022), and surface freshwater ecosystems (Olefeld et al., 2020; Lepère et al., 2013), largely neglecting groundwater. Even the most comprehensive review in the field entirely overlooks groundwater ecosystems (Singer et al., 2021).

This study directly compares the protist community composition and diversity of an oxbow lake with that of shallow groundwater 25 meters from the shoreline. Beyond the obvious lack of light, the shallow aquifer differs from surface water in trophic state, oxygen availability, and the amplitude of seasonal environmental dynamics, which together affect the organisms present. The groundwater habitat poses specific challenges and is typically inhabited by well-adapted organisms that form diverse communities distinct from those found at the surface. However, the consequences of hydrological exchange between surface water and shallow groundwater for protistan community composition dynamics remain unclear, particularly with regard to the proportion of generalist taxa (i.e., those consistently detected in both environments) relative to specialists (i.e., those restricted to either surface or subsurface habitats). In addition to passive dispersal, protists can actively move by gliding or swimming, and some can also attach stably to surfaces. Taxa associated with sediment matrices are likely to be attached or gliding, such as cercozoans and amoeba. Conversely, free-swimming taxa and filter-feeding flagellates are hypothesized to be more abundant in the water column of aquatic habitats. Nevertheless, their feeding mechanisms may be similar and include interception feeding, filter feeding, and grazing.

Based on morphological identification, the largest proportion of groundwater protist communities comprises heterotrophic flagellates, including bodonids, cercomonads, and cryptomonads. The absolute numbers of these organisms depend on the availability of organic matter (Novarino et al., 1997; Risse-Buhl et al., 2013; Loquay et al., 2009). First instances of molecular barcoding identified ciliates as the most abundant taxa in karstic aquifers (Herrmann et al., 2020); however, the variability in rDNA operon numbers among different taxa causes a bias in protistan barcoding, which is a biological limitation (Medinger et al., 2010). Copy numbers have been positively correlated with cell size and should be interpreted with caution, but they can provide useful relative and semi-quantitative information. Beyond the organic load, it is difficult to determine which environmental factors influencing surface communities are important in the groundwater environment. Phototrophic taxa, which play a significant role in the diversity and activity of surface habitats, are not expected to prosper in groundwater and could therefore be useful indicators for surface water infiltration. The pH and mean annual precipitation trends that were shown to influence soil communities (Oliverio et al., 2020) are secondary to site-specificity and the effect of water quality when it comes to wetlands (An et al., 2023). Yet seasonal drivers and a clear annual cycle have recently been studied in brooks and ponds (Simon et al., 2015), and in lakes (Cruaud et al., 2019), where the wet and dry seasons defined community composition. This is consistent with the well-documented seasonal variations in the abundance of freshwater diatoms and dinoflagellates (Sommer et al., 1986). Although the seasonality of protists is pronounced in most ecosystems and likely plays a role in shallow groundwater, it is unlikely to be the most important driving factor. In some cases, anthropogenic stressors were described (Garner et al., 2022), as were the impacts of climate change (Gerasimova et al., 2024). This further underscores the need to establish a baseline for groundwater protists before changes occur.

This study aimed to identify the temporal dynamics and ratios of protist taxa in a backwater and in neighboring shallow groundwater within the wetlands of the Danube Floodplain National Park in Austria. Samples were collected every month for two years, providing sufficient temporal resolution to capture seasonal trends in environmental and compositional dynamics in inland waters (Cruaud et al., 2019; Simon et al., 2015).

Both the total community and the active fraction were characterized using DNA- and RNA-based analyses. DNA-derived 18S rRNA gene sequences represented the total community, whereas RNA-derived 18S rRNA sequences reflected the ribosomal RNA pool of the potentially active community. High-throughput sequencing of the V9 region of the 18S rDNA gene was performed with established primers (Geisen et al., 2019) to characterize the protist community and to enable comparison with results from other 18S-based studies, particularly those from groundwater ecosystems (Herrmann et al., 2020).

We hypothesized the following: (i) a clear distinction between the environmental conditions in surface water and groundwater accompanied by a pronounced difference in the protistan community composition of the two, (ii) lower diversity of groundwater protists than in the surface water, (iii) a dominance of non-photoautotrophic taxa in groundwater, as a result of the specific environmental conditions, and (iv) more pronounced seasonal dynamics in surface water. Furthermore, considering that the Lobau groundwater sampling site is likely to be recharged at a higher rate during the winter months (Dreher et al., 2006), (v) we hypothesized that the similarity of groundwater protistan communities to surface-water communities would be higher in winter.

## 2 Materials and methods

### 2.1 Sampling and study area

**Figure 1:**
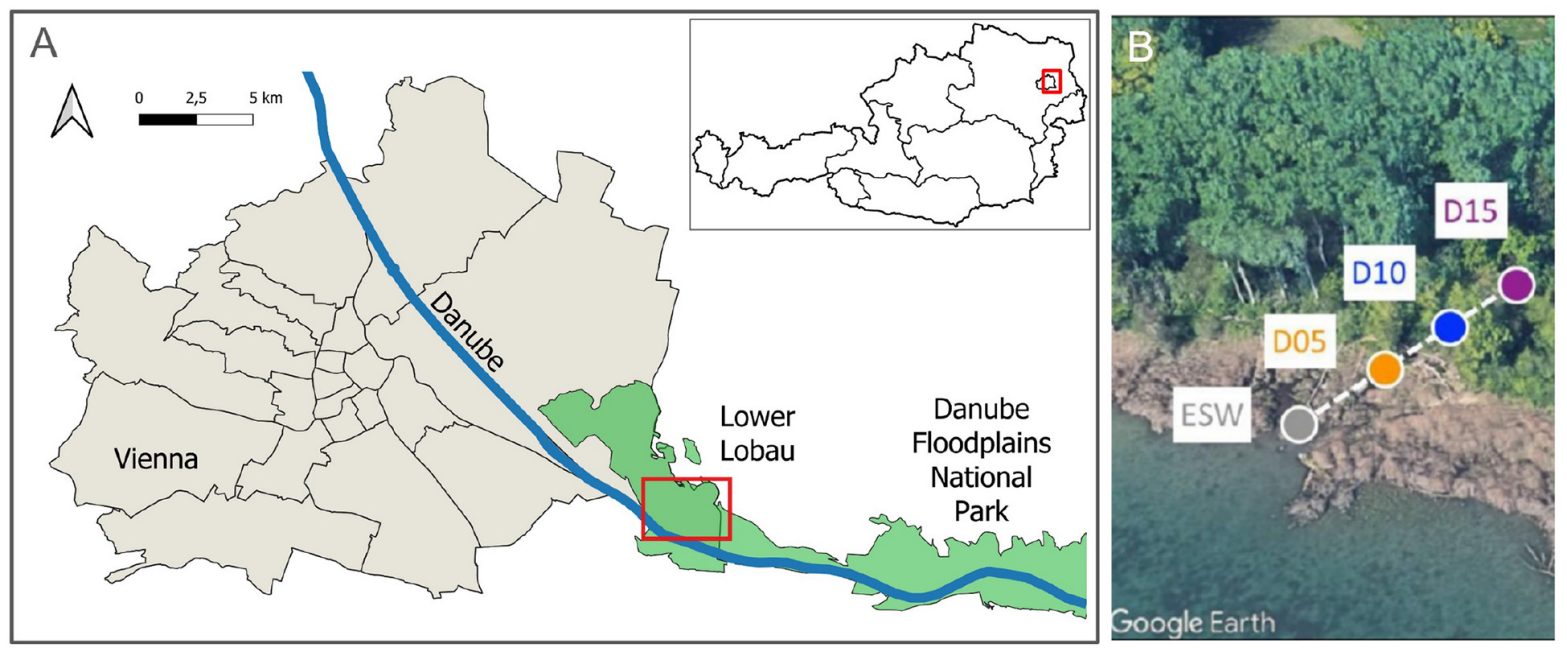
**(A)** Location of the sampling sites in Vienna, Austria. City and state boundaries: © Stadt Wien (data.wien.gv.at, 2025). Rivers and protected areas: © OpenStreetMap contributors. **(B)** Positioning of the Lobau sampling sites. Base imagery in panel B: © Google Earth. Sites D05, D10, D15, and ESW were analyzed for protist communities.

From summer 2020 to summer 2022, surface water and groundwater samples were collected in the Viennese Danube wetlands area of Austria's National Park Lobau. Three groundwater wells and one backwater (Figure 1) were sampled monthly; however, only the water samples from the 10-meter-deep groundwater well D15 and the backwater Eberschüttwasser (ESW) were processed for protist community analysis. These samples represent the extremes of environmental conditions between surface water and groundwater.

The groundwater flow direction and hydraulic gradients between the monitoring wells were determined using a triangulation method based on hydraulic heads expressed in meters above sea level (m.a.s.l.). For each time point, a triangular flow grid was constructed from three groundwater wells (D05, D10, and D15) and, in most cases, the nearby groundwater well D17. Equipotential lines were interpolated and visualized in QGIS, v3.44.4, (QGIS Development Team, 2025). Water flow was determined to be perpendicular to equipotential lines and directed from the maximum to the minimum hydraulic head.

### 2.2 Sample collection

A submersible pump was used to extract groundwater from a depth of 6 meters in well D15. First, the stagnant water was removed, and the well volume was exchanged twice. After pre-pumping, when key environmental variables [temperature, dissolved oxygen (DO), electrical conductivity (EC), and pH] stabilized, they were recorded using field sensors (WTW Multi 3620, Weilheim, Germany). Lake samples were collected at a depth of around 30 cm below the surface. Water samples for hydrochemical analysis were filled directly into 50 mL plastic tubes without any treatment. Water samples for dissolved inorganic carbon (DIC) analysis were filtered on site through a 0.45 μm syringe filter. The same procedure was followed for dissolved organic carbon (DOC) samples with the additional step of adding HCl to reach a pH < 2. Water samples intended for molecular analysis were collected in 10 L carboys and filtered in the laboratory through a 0.22 μm Sterivex filter. The filters were stored at –80 °C until further processing.

### 2.3 Measurement of physico-chemical and microbial parameters

Concentrations of major ions (Na^+^, K^+^, Ca^2+^, Mg^2+^, Cl^−^, ${\text{SO}}_{4}^{-}$) were measured by ion chromatography (Dionex ICS1100 RFIC, Thermo Scientific, Idstein, Germany). Greenhouse gases carbon dioxide (CO_2_) and methane (CH_4_) were measured via laser spectroscopy (Picarro, G2201-i), as were the stable water isotope signatures (δ^18^O, δ^2^H). Dissolved organic and inorganic carbon were quantified with a TOC-L analyzer (Shimadzu TOC-L). The ratio of total and external adenosinetriphosphate (ATP), often used as a proxy for microbial activity, was measured following the Hammes et al. (2010) protocol, with the same adjustments as in Retter et al. (2023). The measurement was performed with the BacTiter-Glo Assay (Promega, USA) in a luminescence plate reader (Promega, Madison, WI, USA). Internal activity, used in the analyses and referred to as ATP, was calculated as the difference between the total and external ATP. The total counts of prokaryotic cells (TCC) were measured with a Cytek^®^ Amnis^®^ CellStream^®^ flow cytometer (Amnis CellStream, Luminex, Austin, TX, USA), after being stained with nucleic acid dye SYBR Green I (Invitrogen, Darmstadt, Germany), using the same settings as Retter et al. (2023).

### 2.4 DNA and RNA extraction, Illumina sequencing and sequence processing

The genetic material was extracted based on a phenol-chloroform extraction protocol optimized for the co-extraction of RNA and DNA (Lueders et al., 2004) with some modification (Lever et al., 2015) to prepare for the 18S rDNA gene amplicon sequencing. The 0.22 μm Sterivex filters, which contained the microbial biomass, were cut into small pieces and put into 2 mL tubes containing zirconia-silica beads (Biospec, Bartlesville, USA), in a 1:1 ratio of 0.1 mm and 0.7 mm size. Next, 160 mol/L NaPO_4_, 3.191 × 10–5 mol/L TNS, and 11.4 mol/L PCI (phenol-chloroform isoamyl alcohol in 25:24:1) were added, and cells were lysed via beadbeating (FastPrep24, MP) for 45 s at 6.5 m/s. The samples were centrifuged (13,000 g and 4 °C) for 4 min, and the resulting supernatant was taken (900 μl) and extracted with 1 volume PCI (11.4 mol/L). After centrifugation, 800 μL of the supernatant was mixed with 0.011 mol/L CI (24:1 chloroform:isoamyl alcohol) and transferred to a “Phase Lock Gel Light” column (Eppendorf, Hamburg, Germany), separating (13,000 rpm, 4 °C, 4 min) the nucleic acids into the upper aqueous phase. The supernatant (650 μl) was mixed with two volumes of PEG buffer (30% polyethylene glycol) during a 30-min centrifugation step for precipitation. The supernatant was removed, and the DNA/RNA pellet was washed with 11.98 mol/L ethanol and cooled at –20 °C. Following a final centrifugation (13,000 rpm, 4 °C, 4 min), the ethanol was removed and evaporated at room temperature (20 °C). Finally, the DNA/RNA was resuspended in 0.01 mol/L EB buffer from the QIAGEN kit. The aliquots intended for DNA amplicon sequencing were stored at –80 °C. The aliquots intended for RNA amplicon sequencing were further processed by first removing the DNA with the TURBO DNA-free Kit (Thermo Fisher Scientific) according to the manufacturer's protocol. The RNA was then converted into cDNA with the RevertAid First Strand cDNA Synthesis Kit (Thermo Fisher Scientific). The extracts were stored at –80 °C until being sent, together with DNA extracts, for amplification and sequencing of the V9 region of the 18S rDNA, with the 1380F/1510R primers, based on the Pjevac et al. (2021) protocol.

The bioinformatic processing of the raw sequence data was done using DADA2 (Callahan et al., 2016) to select the viable amplicon sequence variants (ASV). The processing was performed by the Joint Microbiome Facility (JMF) under the project ID JMF-2301-09. Subsequently, quality filtering was performed, as well as forward and reverse read merging, and further processing of the ASV abundance table by removing the ASVs that could not be classified at the supergroup level or those that were higher-level eukaryotes. Sequence classification was based on the PR2 database (Vaulot et al., 2022).

### 2.5 Statistical analyses

All data were analyzed and visualized in R (R Core Team, 2024; Wickham, 2016). Sampling sites with a sequence count >100 made up 46 DNA-analyzed samples and 43 RNA-analyzed samples. Sampling dates were categorized by seasons based on the month of collection: winter (December–February), spring (March–May), summer (June–August), and autumn/fall (September–November). Species accumulation curves, diversity estimates, and principal component analysis of the environmental parameters were performed using the “vegan” functions under the default settings (Oksanen et al., 2001). Community turnover and seasonal dissimilarities were calculated as the Euclidean distance of central log-ratio (clr) transformed counts using the “microViz” package (Barnett et al., 2021). This was done because of substantial variability in read counts between the two sampling sites, rendering the dataset unsuitable for rarefaction. Clr transformation followed by Euclidean distance calculations corresponding to the Aitchison distance was considered a suitable alternative to Bray-Curtis dissimilarity for compositional data (Gloor et al., 2017). The distribution and spread of the univariate variables were tested and compared between groups according to the assumptions, using the “rstatix” (Kassambara, 2023). Community composition was tested using “microViz” wrappers (Barnett et al., 2021) for the “adonis2” and “betadisper” functions. Unless stated otherwise, values are reported as mean ± standard deviation. The relationship of community composition to significant environmental variables was identified using the “capscale” functions and tested using the “anova.cca” function in the “vegan” package, after filtering out variables with a variance inflation factor (vif) > 10. Seasonality was tested using the combined test from the “seastests” package (Ollech and Webel, 2020), and differential analysis and effect size estimation were performed using the lefse function in the microeco package (Liu et al., 2021; Segata et al., 2011). Core taxa were identified using the “microbiome” package (Lahti and Shetty, 2019), based on the following criteria: after DNA- and RNA-based reads were pooled for each sample from which they were extracted, an ASV needed to be present at least once in a sample and found in more than 75% of samples to be considered a core ASV. This revealed which protists were consistently present (DNA) versus those that were potentially actively functioning (RNA), highlighting dormant/inactive members (high DNA, low RNA) and metabolically key players (high abundance in both or high RNA).

## 3 Results

### 3.1 Sampling site characterization

**Figure 2:**
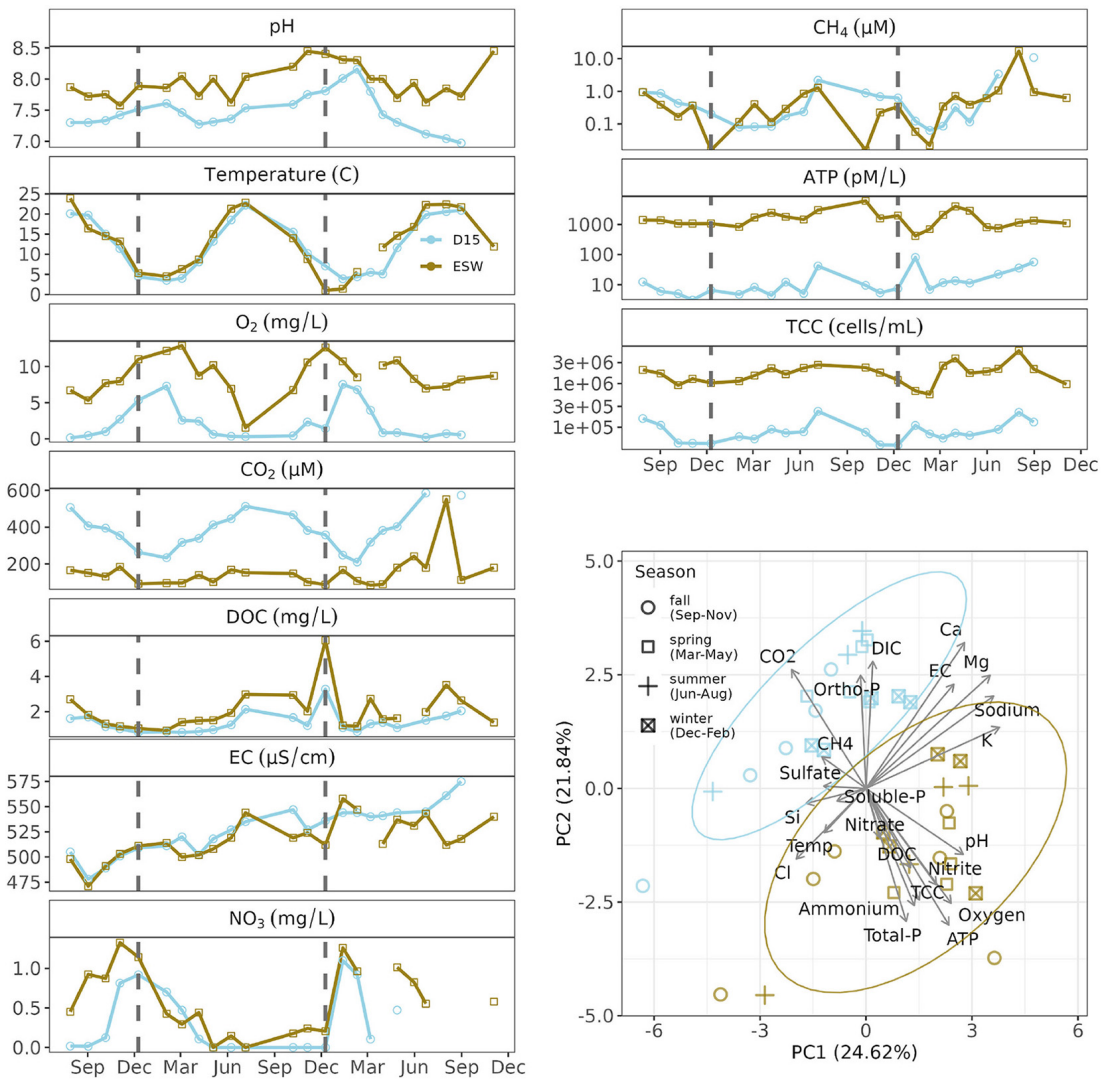
Time series of dynamic environmental variables for summer 2020 to winter 2022. Dashed lines represent the start of new year. Environmental characterization of the surface water and groundwater sampling sites via PCA. Color key: blue corresponding to the D15 groundwater sites, brown to the ESW surface water. ATP, internal ATP [pM/L]; CO2, carbon dioxide [μM], DIC [mg/L]; DO, dissolved oxygen [mg/L]; DOC, [mg/L]; CH_4_, [μM], Electric conductivity [uS/cm]; TCC, [cells/mL]; Sulfate, [mg/L]; Ca, calcium [mg/L]; Mg, magnesium [mg/L], Sodium [mg/L]; K, potassium [mg/L]; Ortho-P, orthoposphate [μg/L]; Si, silicate [mg/L]; Temp, [°C]; Soluble-P, soluble phosphate [μg/L]; Total-P, total phosphorous [μg/L]; Cl, chloride [mg/L]; Ammonium, [μg/L]; Nitrate, [μg/L].

Environmental conditions were shaped by both the aquatic habitat type (surface water vs. groundwater) and the season (Figure 2). The groundwater sampling site, termed D15, was characterized by elevated CO_2_ concentrations, DIC concentrations, sulfate, and ortho-phosphate, compared to the surface water, ESW (t_CO2_ = 7.77***; W_DIC_ = 403**, W_SO4_ = 350**, and W_Ortho − P_ = 455***, Table 1). The microbial activity, measured as intracellular ATP, and prokaryotic cell densities were significantly lower in groundwater (W_ATP_ = 0***, W_TCC_ = 0***), as were the DOC and DO concentrations, pH levels, and ammonium concentration (W_DOC_ = 137***; t_DO_ = –8.7***; t_pH_ = –5.82**, t_NH4_ = –2.5***).

**Table 1:** Values for environmental variables, means, and standard deviations written by sampling site.

**Environmental variable**	**Site ID**	**Value (mean ± sd)**
pH	D15	7.47 ± 0.30
	ESW	7.96 ± 0.27
Temperature [°C]	D15	12.03 ± 6.75
	ESW	13.22 ± 7.13
Dissolved oxygen [mg/L]	D15	2.21± 2.43
	ESW	8.72 ± 2.59
Carbon dioxide [μM]	D15	386.43 ± 104.75
	ESW	154.32 ± 94.13
DOC [mg/L]	D15	1.39 ± 0.58
	ESW	2.05 ± 1.13
DIC [mg/L]	D15	57.28 ± 4.98
	ESW	53.59 ± 4.65
Electric conductivity [uS/cm]	D15	527.23 ± 23.80
	ESW	518.04 ± 20.45
Silicate [mg/L]	D15	2.17 ± 0.41
	ESW	2.02 ± 0.57
Nitrate [μg/L]	D15	0.32 ± 0.4
	ESW	0.59 ± 0.42
Nitrite [μg/L]	D15	0.85 ± 2.17
	ESW	1.80 ± 0.91
Ammonium [μg/L]	D15	10.60 ± 10.39
	ESW	19.60 ± 13.56
Sulfate [mg/L]	D15	27.73 ± 4.73
	ESW	25.94 ± 1.94
Orthoposphate [μg/L]	D15	2.27 ± 1.63
	ESW	0.53 ± 0.47
Soluble phosphate [μg/L]	D15	3.16 ± 1.74
	ESW	2.71 ± 1.24
Total phosphorous [μg/L]	D15	4.39 ± 3.28
	ESW	16.96 ± 20.29
CH_4_ [μM]	D15	7.04 ± 28.04
	ESW	1.14 ± 3.42
Internal ATP [pM/L]	D15	16.67 ± 19.85
	ESW	1745.36 ± 1233.71
TCC [cells/mL]	D15	8.91 × 10^4^ ± 5.38 × 10^4^
	ESW	1.90 × 10^6^ ± 1.07 × 10^6^

Seasonal dynamics in CO_2_, DOC, DO, pH, and temperature were apparent at both sampling sites (Figure 2), but were not significant (Ollech and Webel seasonality test showed all variables to have *p*>0.05). Groundwater temperatures showed a short time lag relative to surface water dynamics but were comparable in amplitude, ranging from 22.5 °C in summer to 1 °C in winter. The gases (CO_2_, DO, and CH_4_) followed a general yearly trend, but with considerable differences in amplitudes. In ESW, the DO was mostly above 6 mg/L (8.72 ± 2.59) except in July 2021 (1.54 mg/L), while groundwater from D15 was predominantly hypoxic (2.21 ± 2.43). Groundwater samples exhibited more dynamic CO_2_ concentrations than surface water samples, with peaks in summer when the concentrations of dissolved O_2_ decreased to hypoxic and anoxic conditions. However, surface water exhibited greater variability in methane levels than groundwater and experienced sudden declines in winter. Dissolved organic matter and pH varied throughout the year, without the clear summer-winter contrast observed in other environmental variables. Nevertheless, a similar temporal trend was observed at both sampling sites.

In winter 2020, the electrical conductivity of the two sites differed little. Groundwater conductivity only stabilized at a higher level than that of surface water toward the end of the study period, in 2022. Values of δ^18^O and δ^2^H in water showed comparable isotope trends (ρ_18O_ = 0.94, ρ_D_ = 0.93) for both sites, but with a higher deuterium excess permil in late summer, fall and winter 2020 (10.2 ± 0.81) than in spring and summer 2021 (9.26 ± 0.43). The groundwater and surface water δ^18^O values were close (Supplementary Figure S1A), and showed that the surface water was strongly influencing the shallow aquifer. In other words, the proportion of surface water infiltrated in the shallow aquifer was high. This is also supported by the differences in water levels between ESW and D15 (Supplementary Figure S1B) and the differences in hydraulic head between D05 and D15 (Supplementary Figure S2). These results indicate that surface water infiltrated the shallow aquifer at nearly all time points and that water flowed unidirectionally throughout the study. However, the gradient strength varied, showing alternating phases of fast and slow infiltration, but without a distinct seasonal trend (Supplementary Figure S2). Isotope measurements for the year 2022 were not available and could not be used for a comparison to the changes in electric conductivity behavior.

Prokaryotic cell densities (TCC) and microbial activity (ATP) exhibited drastically different values and ranges between the two sites. Both measures were lower by two orders of magnitude in groundwater than in surface water (Table 1). Overall, the trends were similar, despite differences in absolute values. The summer peaks in prokaryotic activity and abundance; however, they were clearer in groundwater.

### 3.2 Diversity and richness of protist communities

**Figure 3:**
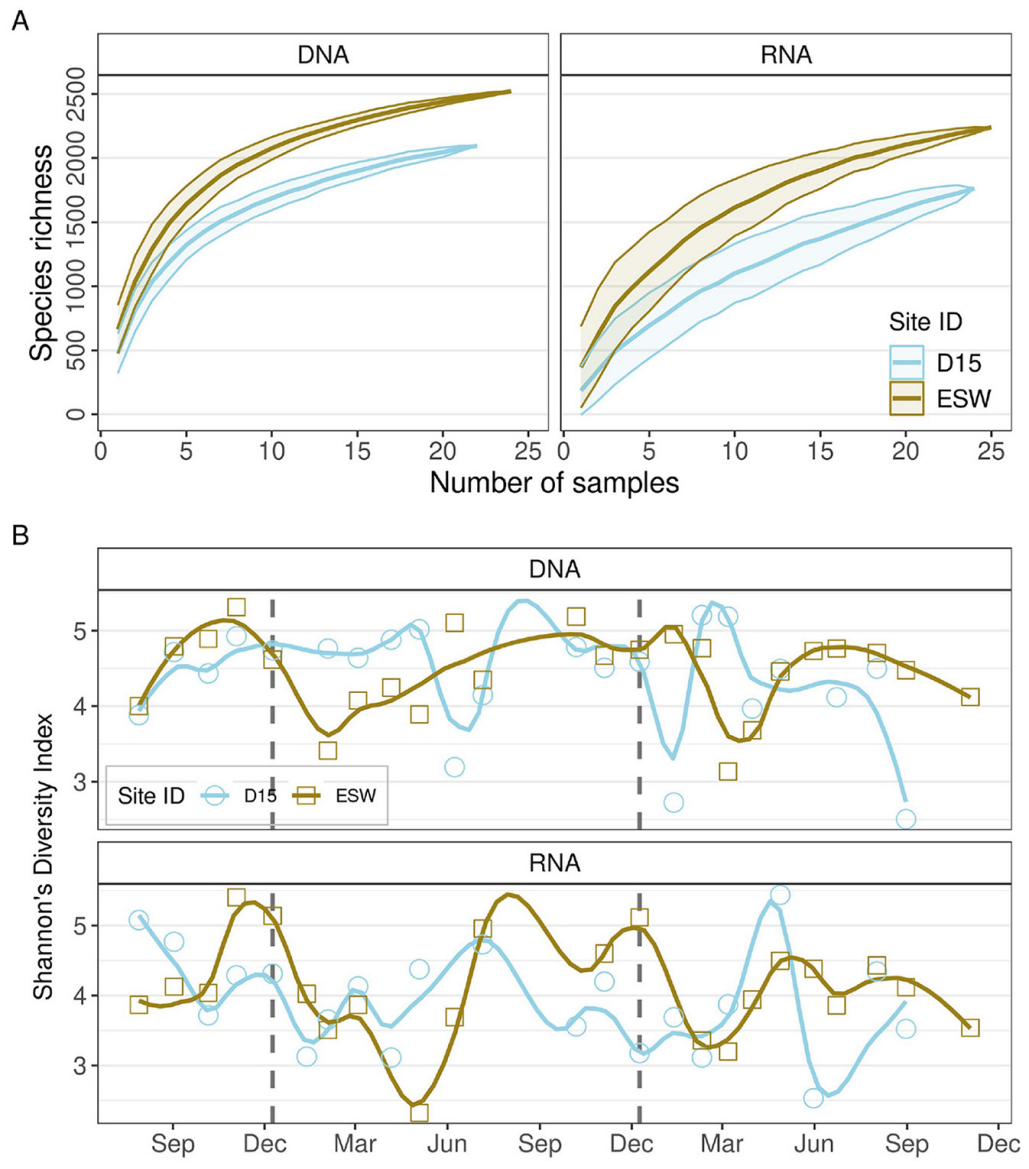
**(A)** Species-accumulation curve for the samples, according to the sampling site and method. **(B)** Protist Shannon's diversity index with a loess fit, according to sampling date and method.

Species accumulation curves indicated that protist communities in neither surface water nor groundwater were fully captured with the current sampling effort, since neither DNA- nor RNA-detected species reached a plateau (Figure 3A). However, compared to surface water, groundwater showed a steeper slope for DNA- and RNA-based barcoding, and the comparison for species richness showed that the DNA-based method detected a slightly greater number of taxa. In DNA- and RNA-derived communities, 3,368 and 3,100 taxa were present, respectively. Not only was the richness of detected ASVs higher in surface water, but the sequence counts also showed pronounced differences by site (DNA: median_(D15)_ = 8,187, median_(ESW)_ = 10,148; RNA: median_(D15)_ = 6,173, median_(ESW)_ = 13,982).

Higher richness in the surface water did not translate into higher Shannon diversity (Figure 3B). There was no difference in Shannon diversity between the surface water and groundwater, but seasonality was significant [*F*_(7, 38)_ = 2.59, *p* = 0.028, adj. *R*^2^ = 0.198], especially for the DNA-derived community, when accounting for the site specificity. Neither season nor site alone could be identified as a significant driver of diversity. The lowest diversity of protists in surface water was observed in spring, while peak diversity was observed in groundwater. The sites were most similar in the winter. The active community exhibited a more dynamic behavior throughout the year, with the highest similarity between sites found in spring.

The seasonality category explained diversity behavior better than any combination of the recorded environmental variables, including those that exhibited seasonal patterns. However, significant trends showed that surface water diversity followed the behavior of ammonium (ρ = –0.68,) and nitrate (ρ = 0.48), and groundwater diversity corresponded to changes in temperatures (ρ = 0.56), dissolved oxygen (ρ = –0.51), pH (ρ = –0.46), CO2 (ρ = 0.58), CH4 (ρ = 0.58), and microbial activity (ATP) (ρ = –0.23).

### 3.3 Seasonal dynamics of protist communities

**Figure 4:**
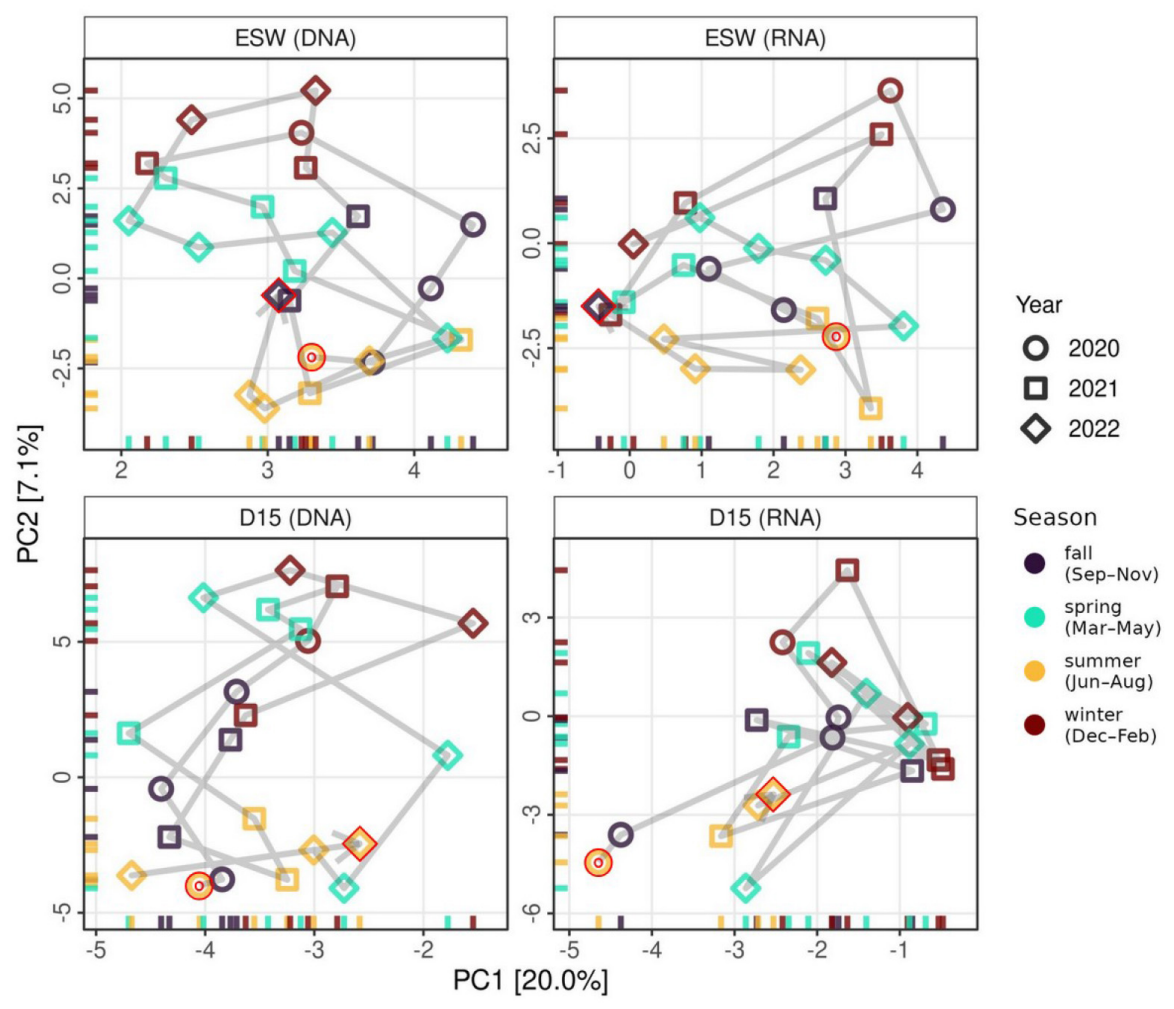
PCA of the protist composition for each sampling site and method used, according to the season and year. The sampling times are represented as a sequential line between the points. The start and end points of each trajectory were highlighted with a red outline.

**Figure 5:**
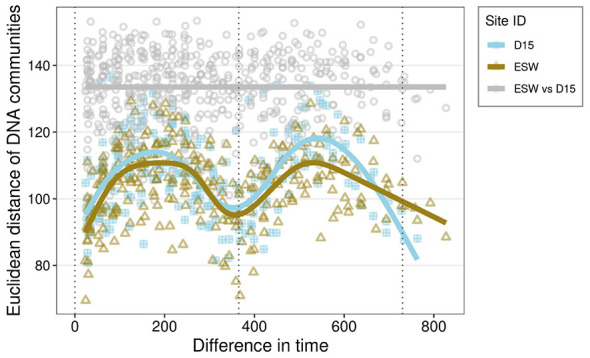
Community pairwise dissimilarity of clr-transformed abundance counts according to Euclidean pairwise time distance in days, colored by sampling site, with the loess fit in corresponding color.

Species turnover of the active groundwater communities was significantly higher than that of the total, DNA-derived communities [F_(1, 41)_ = 6.34, *p* < 0.02], but there was no difference in turnover between the sampling sites. Community composition was significantly different between groundwater and surface water, which was observed in both the DNA [*F*_(1, 44)_ = 14.34, *p* = 0.001, adj.*R*^2^ = 0.25] and the RNA-derived communities [*F*_(1, 41)_ = 6.22, *p* = 0.001, adj.*R*^2^ = 0.13]. There was also a significant difference in community composition between the two habitats based on the presence and absence of taxa [DNA: *F*_(1, 44)_ = 7.32, *p* < 0.01, adj.*R*^2^ = 0.14; RNA: *F*_(1, 41)_ = 5.56, *p* < 0.05, adj.*R*^2^ = 0.12; Supplementary Figure S3]. Seasonal patterns were evident in both sites, both in the total and active communities. Composition followed a yearly cycle transitioning from summer- to winter-specific assemblages, including the autumn/spring transitions (Figure 4). Season alone explained 5.3% of the variance in the total community and, when considered together with habitat type and their interactions, this increased to 34.5% [*F*_(7, 35)_ = 4.16, *p* < 0.001]. The return to the summer composition became even clearer when community dissimilarity over time was examined, with the compositions of summers 2021 and 2022 being more similar to the initial August 2020 composition, and differing markedly from the winter composition (Figure 5). Dissimilarities between sites remained stable throughout the study period and were higher than any within-site dissimilarity. The total community could be tied to seasonally dependent environmental variables such as temperature, microbial activity, and CO_2_ levels [*F*_(3, 39)_ = 5.9, *p* < 0.001, adj.*R*^2^_DNA_ = 0.259]. However, these variables explained less compositional variability than season and site constraints. Active communities were also tied to DOC, NO_3_, and CH_4_ [*F*_(3, 36)_ = 2.37, *p* < 0.001, adj.*R*^2^_RNA_ = 0.10], compared to the 15% variance explained by the season and site [*F*_(7, 32)_ = 1.99, *p* < 0.001].

### 3.4 Community composition

**Figure 6:**
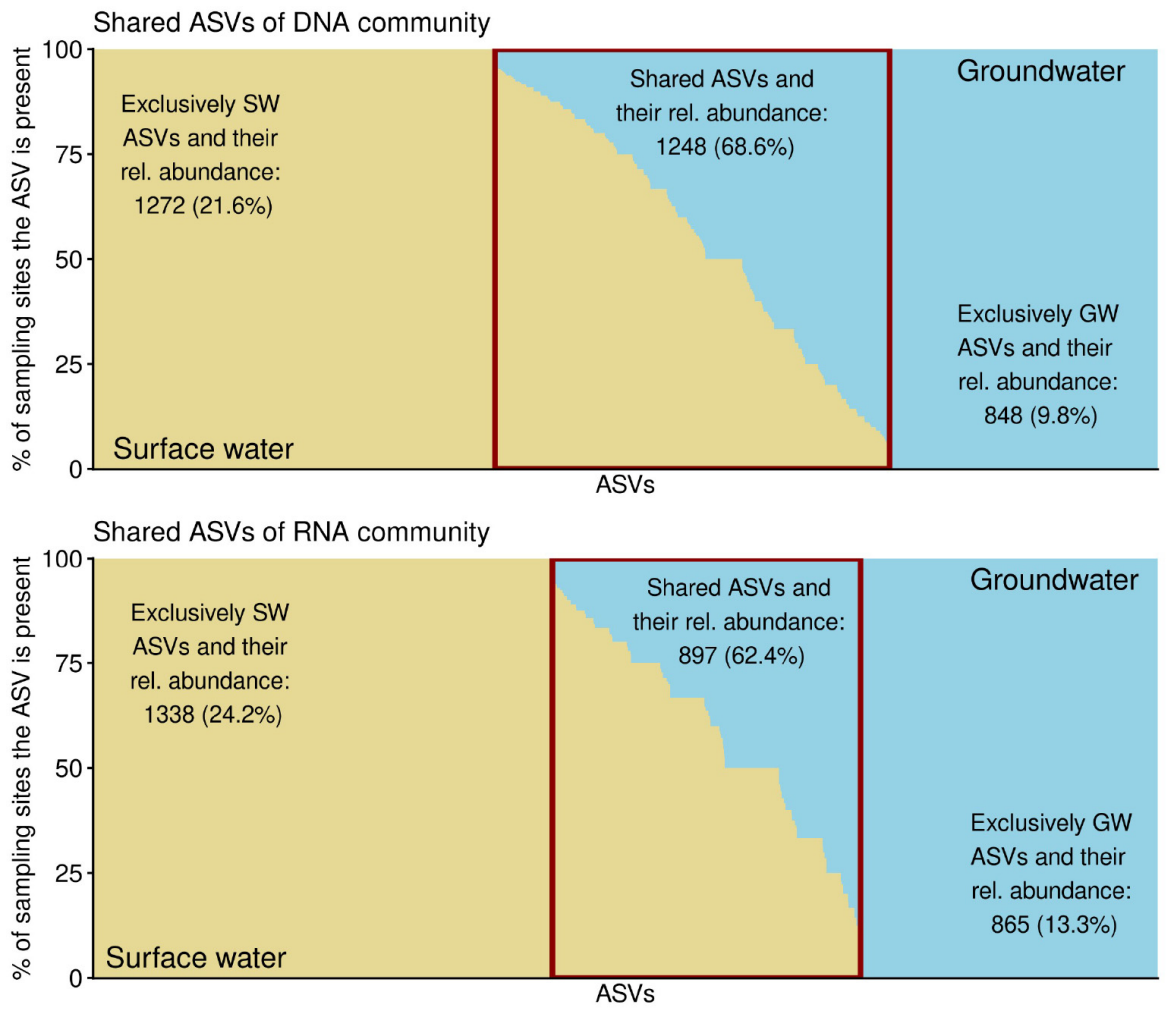
ASV distribution in groundwater and surface water, with red rectangle denoting the shared ASVs. Numbers of taxa and their relative abundances in the habitat they were present in are written for each fraction.

The distribution of ASVs showed that the number of taxa shared between the two sites was not much lower than the number of ASVs specific to each habitat (Figure 6). However, in terms of abundance, the shared ASVs were the more dominant component of the communities. Approximately two-thirds (68.6%) of the total, DNA-derived communities corresponded to ASVs that were shared between surface water and groundwater. A similar pattern was observed for RNA-derived communities, where 62.4% of the relative abundance was attributed to shared ASVs.

An intersection analysis of presence/absence data (Supplementary Figure S4) revealed extensive overlap between DNA- and RNA-based ASVs within both habitats. This shows that a large proportion of taxa detected in the total community are also recurrently detected in the active community. Therefore, after pooling the DNA- and RNA-based ASVs for each sample, core communities were defined for each habitat. Groundwater samples harbored a comparatively small core community (*n* = 46 ASVs), dominated by representatives of *Chrysophyceae* and *Kinetoplastida* (Table S1). Conversely, surface water samples exhibited a substantially larger core community (*n* = 150 ASVs), comprising diatoms (*Bacillariophyta*), *Dictyochophycea*, cryptophytes, chrysophytes, and *Oomycota* (Supplementary Table S1).

Taking into account the relative abundances of gene copy numbers (GCNs), ochrophytes and ciliates were found to be the most abundant protistan divisions at both sampling sites. Higher relative abundances of ochrophytes were observed in total and active communities in surface water [ESW_(DNA)_ = 39.96% ± 10, ESW_(RNA)_ = 50.52% ± 23] than in the groundwater [D15_(DNA)_ = 23.36% ± 18, D15_(RNA)_ = 24.15% ± 27], whereas ciliate GCNs were more prevalent in groundwater, accounting for a substantial proportion of the active community (38.84% ± 23.2). The median ratio of ciliates to ochrophytes was higher in the groundwater samples, but the difference between the two sites was more striking for the active communities [D15_DNA_ = 0.75, ESW_DNA_ = 0.43; D15_RNA_ = 2.7, ESW_RNA_ = 0.36].

**Figure 7:**
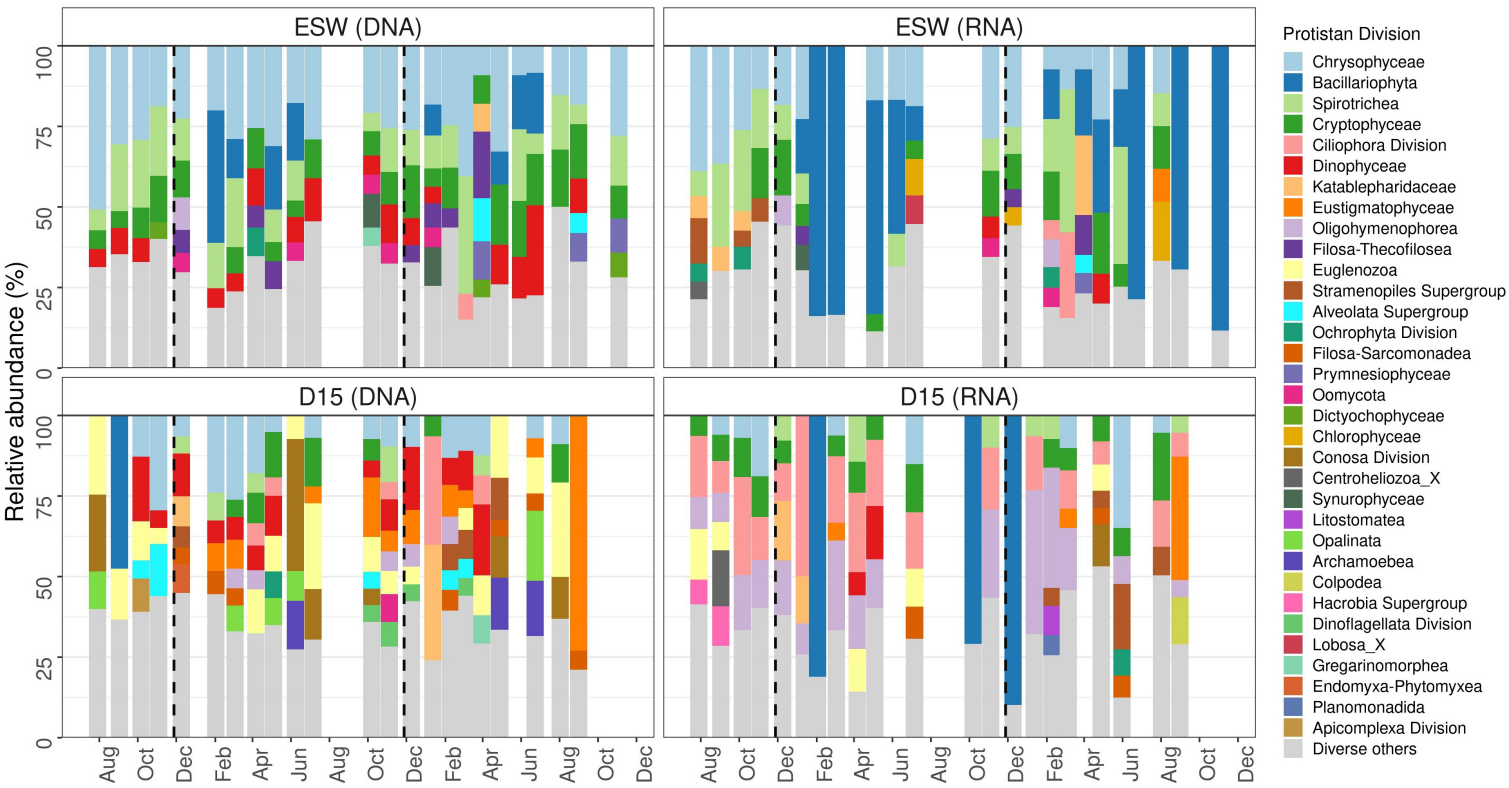
Main protist divisions and their relative abundance in surface water (ESW) and groundwater (D15), according to the barcoding method. Taxa with < 5% of relative abundance are classified under “Diverse others”.

**Figure 8:**
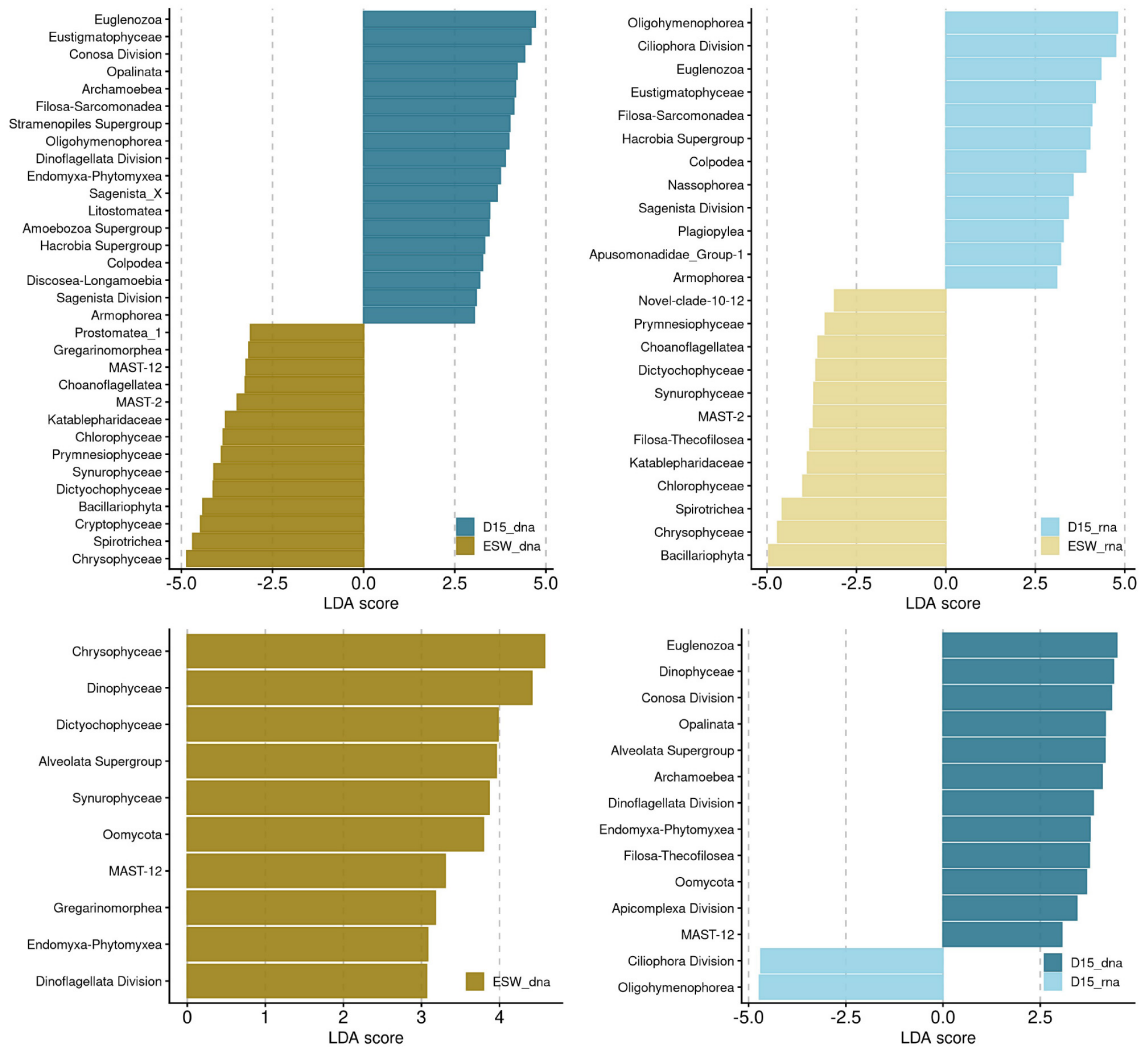
Differential abundance analysis by Linear discriminant analysis Effect Size (LEfSe) of the total and active communities. The bar plots show significant taxa with the effect size >3.

*Spirotrichea* were the most abundant ciliates in both the total and active communities in surface water. whereas a significant portion of the groundwater ciliates were *Oligohymenophorea*, the classified ones mainly belonging to the genus *Tetrahymena* (Figure 7). *Ciliophora* in groundwater were more likely to be unclassified than ciliates found in surface water. *Chrysophyceae* made up a large proportion of the total ochrophyte communities at both sites, but the most dominant *Ochrophyta* in the active communities were *Bacillariophyta*. Both *Chrysophyceae* and *Bacillariophyta*, together with *Dictyochophyceae* and *Synurophyceae*, showed a stronger inclination toward surface water, whereas *Eustigmatophyceae* could be designated as groundwater ochrophytes (Figure 8). The *Bacillariophyta* in question were mainly *Navicula* and *Staurosira*. Flagellates, such as cryptophytes (*Prymnesiophyceae* and *Cryptophyceae*), chlorophytes, choanoflagellates and *Katablepharidaceae* were associated with surface water. In terms of presence, *Cercozoa* were present in both surface water and groundwater. However, surface water was characterized by the cercozoan *Filosa Thecofilosea*, while groundwater had *Filosa Sarcomonadea*, the prevalent class of *Cercozoa*. Although *Filosa Thecofilosea* were present only in low abundance in groundwater, they predominantly comprised active taxa. Other taxa that were significantly more prevalent in groundwater, in terms of both presence and activity, other than the mentioned ciliates and *Filosa-Sarcomonadea*, were *Euglenozoa* and *Apusomonadidae* Group 1.

Five sampling time points characterized by sequence counts drastically different from the general distribution (30–40 sequence counts) were considered separately and showed different compositions compared to the remaining samples (Supplementary Figure S5). The taxa that dominated these few samples were usually rare taxa that were only sporadically present in the rest of the samples. Most notably, the groundwater composition in March 2022 was dominated by *Gregarinomorphea*, which was absent from most other sampling points.

### 3.5 Seasonal trends of individual taxa

**Figure 9:**
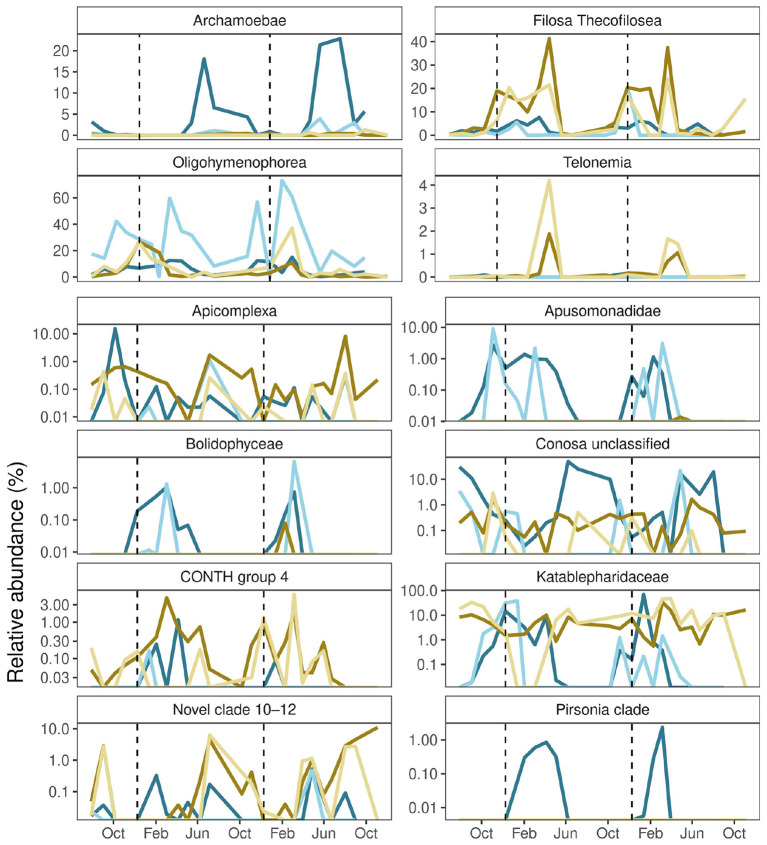
Seasonal dynamics of protistan taxa in groundwater and surface water over two years. Relative abundances (%) protistan classes which showed significant differences between seasons shown across sampling dates. Line colors correspond to different sampling sites and extraction methods. Color key: blue corresponds to the D15 groundwater sites, brown to the ESW surface water, lighter shades represent RNA extracts, and darker shades represent the DNA extracts. Dashed vertical lines indicate the year start.

Associations of taxa to specific yearly periods were present for both winter and summer (Figure 9). *Archamoebea* and unclassified *Conosa* showed an inclination toward summer peaks in groundwater, while their abundances remained stable in surface water. *Telonemia* abundances peaked in early summer in surface waters, whereas unclassified *Apicomplexa* and protists of Novel clade 10–12 reached their surface water peaks in autumn. Winters and springs were optimal periods for *Filosa Thecofilosea* in both groundwater and surface water. However, *Katablepharidaceae* remained stable in surface water throughout the year, while still exhibiting winter peaks in the total and active groundwater communities. Other groundwater-specific seasonal trends were observed for *Pirsonia* clade, as well as for *Apusomonadidae* Group 1 and *Bolidophyceae*, both of which showed a lag in the active community compared to the total community. CONTH 4 showed groundwater peaks that followed the winter surface-water peaks, but these had a limited effect on active groundwater communities. Lastly, *Oligohymenophorea* showed pronounced winter peaks in surface water, but experienced more drastic variability in active groundwater communities.

## 4 Discussion

The scarcity of studies on groundwater protists, coupled with a lack of research employing state-of-the-art molecular techniques, leaves a large gap in our knowledge regarding subsurface eukaryotic communities and their dynamics. This study provides the first overview of groundwater protistan communities in the surface waters and groundwater of the Danube Floodplain National Park in Austria by sequencing the V9 region of the 18S rDNA marker gene. The motivation behind this study was to investigate the transition between surface-water and groundwater habitats and to determine the hypothesized site-specificity, i.e., the impact of factors such as lack of light and limited resources on the composition, functionality, and diversity of protist communities. To our knowledge, this is the first study to focus in detail on the composition and turnover of the protist community in a groundwater habitat and a nearby surface-water habitat over two hydrological seasons. Despite the small-scale and geographical limitations of the study, we believe that the observed compositional dynamics and diversity of protist communities represent benchmark values for other hydrologically connected surface water-groundwater systems with comparable environmental conditions. As such, our findings are relevant beyond the specific study site.

### 4.1 Site specificity and environmental filtering

The shallow groundwater monitoring site in the Danube wetlands was selected as a suitable location to examine the effects of seasonality and surface water on protistan communities. Trends in water levels and stable water isotope signatures indicate groundwater recharge by surface water throughout the year (Supplementary Figure S1). Depending on the difference in hydraulic heads, it is assumed that the time taken for water to travel from the backwater to the shallow groundwater site is 2–4 weeks for a distance of 25 m (Dreher et al., 2006). Continuous hydrological connectivity was supported by predictable patterns in temperature, dissolved oxygen, and carbon dioxide throughout both years (Figure 2). Summer periods were characterized by higher temperatures in both surface water and groundwater, promoting microbial activity and growth. This resulted in increased respiration rates and declining oxygen levels, as well as the release of CO_2_, in a manner previously observed at this monitoring site (Dreher et al., 2006). Depleted nitrate levels accompanied by methane spikes indicated periods of reduced oxygen levels in the surface water sediments and in the aquifer in the summer. These conditions began to change toward a more oxygenated habitat only in late winter.

For the protistan community analysis, the contrasting trends of continuous changes in most redox species, as well as the differences in electrical conductivity and DOC over the two years of the study, were considered. Given the differences in conductivity and the spikes in organic matter between the two years, sudden recharge events and the subsequent disturbances could contribute to protistan community dynamics. In this case, the year of sampling would be a useful predictor. However, this was not observed, since the taxonomic differences between groundwater and surface water sites remained stable throughout all seasons, for both years (Figure 4). Total and active communities were both site-specific in composition, indicating that community changes were distinct and could be detected in the DNA extracts, regardless of their ability to be active in groundwater. Whether this is due to selective attenuation based on size, shape, or other dispersal-related processes requires assessment in future studies. At present, it does not appear to be due to periods of increased recharge. Additionally, most season-specific taxa showed similar behavior in both years, despite differences in recharge probability.

The consistency of community trajectories across two consecutive years, together with the recurrence of seasonal patterns, suggests that the observed dominant temporal signals reflect stable, habitat-specific processes rather than short-term stochastic events. While monthly sampling may not fully capture rapid shifts in protist community composition during highly dynamic phases, such as spring (Šimek et al., 2018; Kavagutti et al., 2023; Mukherjee et al., 2024), the monthly resolution provides sufficient data to support conclusions regarding seasonal structuring, site specificity, and the relative importance of biogeochemical drivers in shaping protistan communities in shallow groundwater.

In shallow aquifers, it is difficult to distinguish the effects of seasonal temperature-dependent processes from those driven by seasonal recharge. However, we did not detect a relationship between protist community patterns and electrical conductivity, which can serve as a proxy for surface-water infiltration. Instead, variations in protist community was associated with CO_2_, CH_4_, and NO_3_ concentrations, suggesting a closer link to seasonal biogeochemical processes that may only be indirectly influenced by recharge dynamics. Ultimately, the temporal community profile remained stable, returning to its initial state after each yearly cycle. This implies that, despite connectivity between the two sites, whether continuous or sporadic, the specificity of the shallow groundwater habitat was likely an important abiotic factor shaping protistan communities during the study period. Our hypothesis that the communities in the two sites would be more similar in winter was not proven. Our theory that seasonal dynamics in groundwater communities are less pronounced than in surface-water protists was also refuted. The pronounced seasonality observed at this groundwater site could be attributed to its shallow depth. This results in a close link between surface temperature fluctuations and those in the surrounding soil, even in the absence of direct groundwater recharge. The upper soil layers would be a source of bioavailable dissolved organic matter, in comparison to the refractory DOM in deeper soil layers (Kaiser and Kalbitz, 2012). Rising temperatures, in combination with readily available DOM, can stimulate microbial growth and activity and cause shifts in the rates of biogeochemical processes during the summer months (Brielmann et al., 2009). Although the presence of phototrophic protist taxa in groundwater supports the idea of continuous connectivity to surface water, the seasonality of protistan communities in groundwater is likely exacerbated by recharge-independent seasonality.

### 4.2 Groundwater-specific protist taxa

Compared to surface water, the groundwater harbored a smaller core community, suggesting that only a limited number of taxa were capable of sustained survival in shallow groundwater. The recurrent detection of kinetoplastid taxa is consistent with previous reports from groundwater systems (Novarino et al., 1997). Their small cell size, planktonic lifestyle, and ability to survive under conditions of low prey availability (Boenigk and Arndt, 2002) likely facilitate their continuous presence at our groundwater site. However, considering the documented bias against kinetoplastids in studies relying on universal eukaryotic primers (Mukherjee et al., 2015), their contribution may be underestimated in terms of relative sequence abundance. Nevertheless, their consistent detection across samples suggests stable persistence within the groundwater community. Overall, these patterns indicate that persistence in groundwater may be influenced by strong ecological filtering, favoring small, free-living, heterotrophic taxa that are adapted to oligotrophic and light-deprived conditions. In line with this interpretation, taxonomic groups forming the core community in surface water, such as diatoms, dictyochophytes, and oomycetes, were absent from the groundwater core community. This pattern is consistent with the ecological requirements of these groups, which typically depend on light availability, higher nutrient concentrations, or particle-associated habitats, conditions that are prevalent in surface waters but strongly limited in shallow groundwater environments.

In contrast to the highly persistent and specific, but not highly abundant taxa, results suggested that the taxa shared between the two habitats contributed substantially to the community composition of both sites (Figure 6). This overlap may be facilitated by the proximity of the water bodies and their continuous hydrological connectivity. Organisms can disperse between surface water and groundwater, with surface water serving as a source of taxa while remaining subject to habitat filtering and seasonal dynamics discussed in the previous section. This could also be related to a greater abundance of generalists compared to specialists in microbial communities (Hernandez et al., 2023).

Site-specific community composition was primarily evident in differences in the ratios of ciliates and ochrophytes. Most groundwater-specific taxa have cilia for locomotion (such as *Oligohymenophorea, Colpodea, Nassophorea*, and *Armophorea*). However, this should be interpreted with caution due to potential molecular biases, particularly the pronounced variation in rRNA gene copy numbers among ciliates (Medinger et al., 2010). Additionally, samples were not pre-filtered before nucleic acid extraction, which may increase the representation of larger-bodied protists with high GCNs. However, our study used the same sampling, extraction, and sequencing protocols for groundwater and nearby surface water. This enables comparison of taxa across habitats and identification of temporal variation within them. Therefore, while we cannot infer the exact number of ciliates, the results indicated that *Ciliophora*-associated genes were more prevalent in groundwater than in surface water. Additionally, although *Ciliophora*-associated genes were strongly represented in the groundwater samples, the dominant taxa were still flagellated protists. Amoebae and ciliates accounted for a smaller proportion of all taxa. This is consistent with previous reports on groundwater protistan communities (Risse-Buhl et al., 2013; Loquay et al., 2009). A lack of ciliates could partly be a result of aquifer geology and pore size, as well as trophic interactions (Novarino et al., 1997). Given that flagellates dominate in sand aquifers (Novarino et al., 1997). The karstic groundwater system is predominantly inhabited by ciliates (Herrmann et al., 2020). The presence of ciliates and their more balanced ratio with flagellate gene copies in the shallow aquifer may be due to the mixed coarse gravel, sand, and silty-clay layers (Danielopol, 1989). The relationship between aquifer characteristics and protist community composition remains to be further explored and confirmed in future studies.

Regardless of aquifer characteristics, it is unlikely that groundwater habitats would provide sufficient trophic resources to sustain a community dominated by ciliates and amoebae over time. These protists require abundant bacterial prey for survival, but the biomass of these bacteria is significantly lower in groundwater than in surface water (Griebler and Lueders, 2009). However, the presence of ciliates in the shallow groundwater cannot be dismissed, as presence/absence analyses, which mitigate molecular bias, also reveal two distinct protistan communities in the two habitats (Supplementary Figure S3). Future studies should aim to quantify all members of the microbial food web to mitigate molecular bias and better understand the conditions under which such taxa persist and remain active in subsurface ecosystems.

The study focused on the planktonic fraction of the protistan community and excluded attached or biofilm-associated taxa. This limitation may also explain the high abundance of ciliates in the free-living groundwater community. In karstic aquifers, ciliates are known to dominate under anoxic conditions, whereas cercozoans become competitive only as oxygen levels increase (Herrmann et al., 2020). A similar pattern was observed at our study site, where the cercozoan species *Filosa thecofilosea* was most abundant in winter and early spring, corresponding with *Oligohymenophorea* abundances and sufficient oxygen levels (Figure 9). Given the high representation of ciliate species in both the DNA- and RNA-derived communities, as well as their well-established bioindicator potential (Foissner, 2004; Stoeck et al., 2018; Kulaš et al., 2021), and the site's proximity to urban influences, long-term monitoring should be considered. In particular, the detection of *Tetrahymena*, which are associated with high saprobic conditions (Foissner, 2016), should be examined.

Other groundwater-specific taxa included those previously reported in other aquifers or soils, such as *Apusomonadidae* and euglenids, which were found to survive even in aquifers with elevated nitrate and chloride levels (Loquay et al., 2009), as well as soil-abundant Cercozoan classes (Oliverio et al., 2020).

The hypothesized scarcity of phototrophic taxa in groundwater was not immediately evident due to the relatively high proportion of ochrophytes in the RNA-derived communities. This group comprises great functional diversity, including obligate heterotrophs, phototrophs, and facultative mixotrophs. Trophic strategies within this group are often context-dependent (Jirsová and Wideman, 2024). Several ochrophyte lineages detected in this study, including *Chrysophyceae, Eustigmatophyceae*, and *Bacillariophyta*, have all shown mixotrophic potential under suboptimal conditions, as long as DOC levels were sufficiently high (Tuchman et al., 2006; Villanova et al., 2022; Lie et al., 2018). While amplicon-based data do not allow unambiguous assignment of trophic modes, the taxonomic composition suggests that heterotrophy was the dominant trophic guild in groundwater, alongside the presence of taxa with mixotrophic and parasitic potential (Figure 9, particularly *Apicomplexa*). Overall, this supports the hypothesis of general heterotrophic dominance in groundwater, whereas the sporadic presence of taxa with mixotrophic potential may reflect the site's transitional nature, driven by continuous connectivity to surface water.

Unclassified members of the *Conosa* group showed a notable increase in relative abundance in the DNA-derived communities in summer. However, their representatives in the RNA pool were mainly observed in oxic periods, consistent with previous reports for some members of the group (Cavalier-Smith, 1998). While it is tempting to attribute the differences in the DNA- and RNA-derived community to dispersal from the surface, they could also be a consequence of pronounced cyst formation in summer, a common strategy among protists under suboptimal conditions. On the other hand, the lag in RNA compared to DNA spikes in winter-specific taxa, such as *Bolidophyceae* and *Apusomonadidae*, could suggest that these protists are introduced to the groundwater from an external source. This is because both taxa appeared in total communities in early winter, but their RNA spikes were only observed a month later. Alternatively, while RNA-derived communities are frequently used as proxies for activity, the abundance of ribosomal RNA does not directly equate to metabolic activity. The rRNA concentration may also be influenced by taxon-specific rRNA turnover rates and by ribosome pool maintenance during dormancy phases, which enables rapid shifts in metabolic functions with changing conditions (Blazewicz et al., 2013). Accordingly, the RNA-derived patterns partly reflect direct activity, but partly differences in community metabolic responsiveness. Lastly, we found evidence of taxa that exhibited divergent seasonal behavior between the two sites, particularly Novel clades 10 and 12. Their abundance peaked in summer in surface waters, implying an origin outside the groundwater system. The current limited knowledge of the ecology of this almost exclusively freshwater planktonic clade does not yet offer conclusive insights.

### 4.3 Diversity

The protist diversity in different habitats spans a wide range of Shannon diversity values. Combined with site-specific environmental differences, this could provide grounds for hypothesizing that diversity patterns differ between freshwater surface and groundwater habitats. However, subsurface conditions present unique challenges and are just as likely to support a diverse community, although one with lower total abundance and biomass. Shallow groundwater protistan richness was indeed slightly lower than in surface water, but differences in Shannon diversity were not significant. The higher richness of surface water may reflect greater external inputs of organisms, but it could also be the result of sequencing bias, since higher protist biomass in surface water could translate to an increased sequencing depth (Schenk et al., 2019). Cell quantification in eukaryotes was beyond the scope of this study; however, it should be addressed in future research at these sites. This research should focus on the dynamics between eukaryotes and prokaryotes and the relationships between the biomass of these two groups, as was done in Karwautz et al. (2022). For this study, therefore, biodiversity indices that account for evenness may provide a more robust estimate of amplicon diversity than richness alone.

One of the few studies addressing protist diversity in transition zones between high-energy systems and nutrient-poor habitats was conducted in a cave at the inflow point and at a site characterized by stable, light-free, oligotrophic conditions. The study reported higher Shannon diversity at the point of surface water inflow, where photosynthetic protists and organic matter were present (Gogoleva et al., 2024). As a continuous hydrological connection cannot be ruled out, a similar interpretation may apply to our study site, which could be considered an equivalent of the cave system in terms of being a transitional zone with sustained environmental variability and opportunities for colonization. This could explain why the diversity of groundwater at our sampling site did not differ substantially from that of surface-water communities. In future studies, it would be advantageous to include a deeper, more distant groundwater site that may harbor distinctly lower-diversity assemblages due to more stable, oligotrophic conditions.

Seasonal dynamics in DNA-derived community diversity were similar across the two sites, particularly in December 2020, further supporting the hypothesis of increased connectivity during this period. However, while diversity calculated from DNA extracts converged in winter, community diversity based on RNA extracts showed the opposite pattern, with greater divergence in winter. Considering a lack of clear delay in groundwater diversity in active communities, and the limited comparability between the trends of the two sites, decoupled diversity dynamics between the two sites were more likely than a connection between them. The differences in the diversity drivers of active protistan communities supported this conclusion. While our two-year study was sufficient to capture stability in community composition, longer-term monitoring may be necessary to elucidate diversity dynamics and their dependence on food web interactions.

## 5 Conclusion

Our study of protistan communities in the Danube Floodplain National Park in Vienna, Austria, provides a detailed characterization of the composition and diversity of the community across a surface water-to-groundwater gradient. This was achieved by using molecular barcoding to analyze total DNA- and active RNA-derived protistan communities. Monthly sampling over two hydrological years revealed distinct and robust compositions and turnover in protistan communities in surface water and groundwater. With respect to diversity, we showed that RNA-based protistan communities in groundwater exhibited seasonal dynamics similar to those in surface water. Functionally, the groundwater community was dominated by heterotrophic protist lineages and taxa with known mixotrophic potential, including ciliates. Groundwater protist diversity was associated with prokaryotic activity and redox conditions, suggesting the need for future screening of food web interactions. This study is among the first to adopt a molecular approach to analyzing protist communities in groundwater. Long-term monitoring and the inclusion of attached communities will be essential for improving our understanding of protist ecology in groundwater.

## Data Availability

The data presented in this study are publicly available. The data can be found here: https://www.ncbi.nlm.nih.gov, accession PRJNA1366129.
