# Supplementary material for: Dynamic changes in protist community composition along a surface water-groundwater transect in the Danube wetland Lobau, Vienna, Austria
Source: Front Microbiol. 2026 Mar 12;17:1749803. doi: 10.3389/fmicb.2026.1749803 (PMC13017956; doi:10.3389/fmicb.2026.1749803)
Supplement: Supplementary file 1 [file Data_Sheet_1.pdf]

Supplementary Material

1 SUPPLEMENTARY TABLES

Table S1: Number of ASVs identified as core taxa in groundwater (GW) and surface water (SW). Taxa marked as “not core” were not identified as part of the core community in the respective habitat, but were core taxa in the other. Taxonomy represents the lower taxonomic level they were identified to.

Taxonomy	Number of core ASVs in GW	Number of core ASVs in SW
Allapsidae Family	1	not core
Allapsidae_X_sp.	1	1
Alveolata Supergroup	2	1
Arcuospathidium_namibiense	1	1
Bodonidae_X_sp.	1	not core
Chrysophyceae_Clade-C Family	1	1
Chrysophyceae_Clade-E_X_sp.	2	1
Chrysophyceae_X Order	5	12
Ciliophora Division	2	5
Cryptomonas Genus	1	2
Cryptomonas_platyuris	1	1
Dinophyceae Class	1	3
Diplophrys_sp.	1	not core
Euglenida Order	1	not core
Euglenozoa Class	1	not core
Eustigmatophyceae_XX Family	1	1
Hacrobia Supergroup	1	not core
Kinetoplastida Order	2	not core
Litostomatea Class	1	not core
Litostomatea_XX Family	1	not core
Mayorella Genus	1	not core
Nannochloropsis Genus	1	not core
Neobodo_designis	2	not core
Neobodonid Family	1	not core
Ochromonas Genus	1	not core
Orthodonellidae_X_sp.	1	not core
Pentapharsodinium_tyrrhenicum	1	not core
Prokinetoplastidae_X_sp.	2	not core
Rhogostoma-lineage Family	1	not core
Rhynchobodo_sp.	2	not core
Sagenista Division	1	not core
Sandonidae Family	1	not core
Stramenopiles Supergroup	2	5
Tetrahymenida_X_sp.	1	not core
Achnanthidium_minutissimum	not core	1
Ankistrodesmus_fusiformis	not core	1
Aphanomyces_sp.	not core	1

Taxonomy	Number of core ASVs in GW	Number of core ASVs in SW
Araphid-pennate Family	not core	2
Asterionella_formosa	not core	1
Basal_Cryptophyceae-1_X_sp.	not core	4
Cercomonas Genus	not core	1
Chlamydaster_sterni	not core	1
Chlamydomonas Genus	not core	1
Chlamydomonas_acidophila	not core	1
Choanoflagellatea Class	not core	1
Choreotrichida Order	not core	2
Chrysophyceae_Clade-B2 Family	not core	1
Chrysophyceae_Clade-E Family	not core	2
Coleps_nolandi	not core	1
Cryptomonas_borealis	not core	1
Cryptomonas_marssonii	not core	1
Cryptomonas_obovoidea	not core	1
Cryptomonas_paramecium	not core	1
Cyclotella Genus	not core	1
Cyclotella_distinguenda	not core	1
Cyclotella_meneghiniana	not core	1
Cymbella_affinis	not core	1
Desmodesmus_communis	not core	3
Dinobryon_divergens	not core	1
Dinobryon_sociale	not core	1
Dinoflagellata Division	not core	1
Eolimna_minima	not core	1
Euglypha_rotunda	not core	1
Eustigmatophyceae_XXX_sp.	not core	1
Fragilaria Genus	not core	1
Gomphonema_affine	not core	1
Goniomonas_truncata	not core	1
Halteria_grandinella	not core	1
Hypotrichia Order	not core	2
Katablepharidales_XX_sp.	not core	3
Lagenidium_caudatum	not core	1
Lagenidium_giganteum	not core	1
Mallomonas Genus	not core	1
Mallomonas_akrokomos	not core	1
MAST-12_XXX_sp.	not core	1
MAST-12C_XX_sp.	not core	3
MAST-2C_XX_sp.	not core	1
Monas_sp.	not core	2
Monocystis_sp.	not core	2
Monosigidae_Group_O_X_sp.	not core	1
Navicula_cryptotenella	not core	1

Taxonomy	Number of core ASVs in GW	Number of core ASVs in SW
Navicula_radiosa	not core	1
Ochrophyta Division	not core	6
Oxnerella_micra	not core	1
Oxytrichidae Family	not core	1
Pedinella_sp.	not core	3
Pedinellales Family	not core	4
Pedinellales_X_sp.	not core	2
Pelagostrombidiidae_X_sp.	not core	2
Peregrinia_clavideferens	not core	1
Peridinium_willei	not core	1
Peronosporales Family	not core	1
Phytopythium Genus	not core	1
Plagioselmis_nannoplanctica	not core	1
Prorocentrum_micans	not core	1
Prorocentrum_sp.	not core	1
Pseudopedinella_elastica	not core	1
Pseudostaurosiropsis_sp.	not core	1
Punctastriata_sp.	not core	1
Pythiaceae_sp.	not core	1
Pythium Genus	not core	2
Raphid-pennate Family	not core	2
Rhodomonas_sp.	not core	1
Rhynosporidae_Desmocystidianae Family	not core	1
Rimostrombidium_D_sp.	not core	1
Scuticociliatia_2_X Family	not core	1
Sellaphora_pupula	not core	1
Sphaeropleales_X Family	not core	1
Staurosira Genus	not core	1
Stephanodiscus Genus	not core	1
Strobilidiidae_C_X_sp.	not core	1
Strobilidium_E_sp.	not core	1
Strombidiida Order	not core	1
Synedra_ulna	not core	1
Synura_spinosa	not core	1
Synurales_XX_sp.	not core	1
Teleaulax_amphioxeia	not core	2
Thaumatomonas_coloniensis	not core	1
Thraustochytriaceae_X_sp.	not core	1
Trinema_lineare	not core	1

2 SUPPLEMENTARY FIGURES

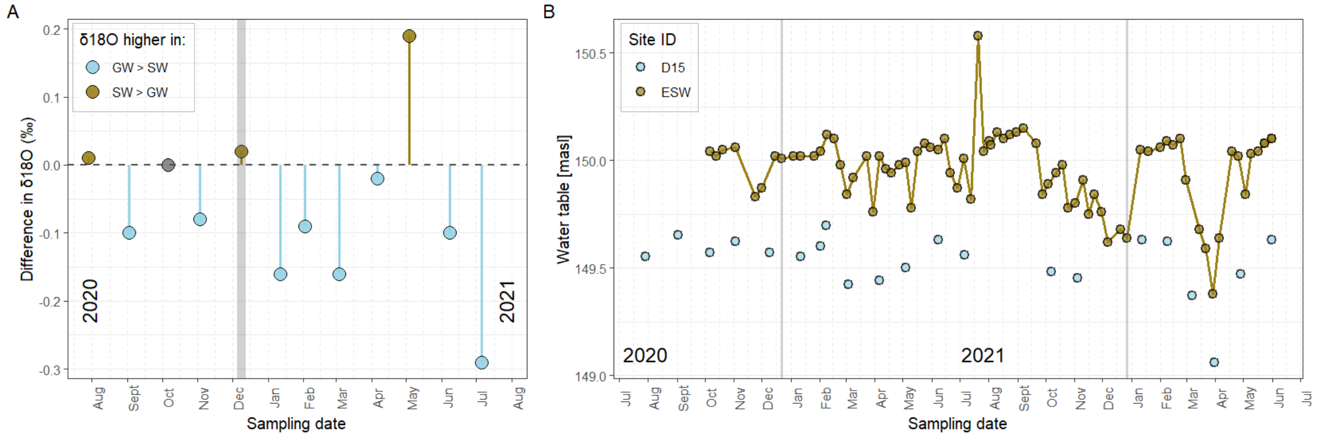

Figure S1: **Left.** Differences in δ^{18}O values between surface water ESW and groundwater site D15, for period summer 2020-summer 2021. **Right.** Water levels of surface water and a nearby groundwater monitoring well in meters above the Adriatic, for period summer 2020 to summer 2022. Start of a new year is marked with a gray horizontal line.

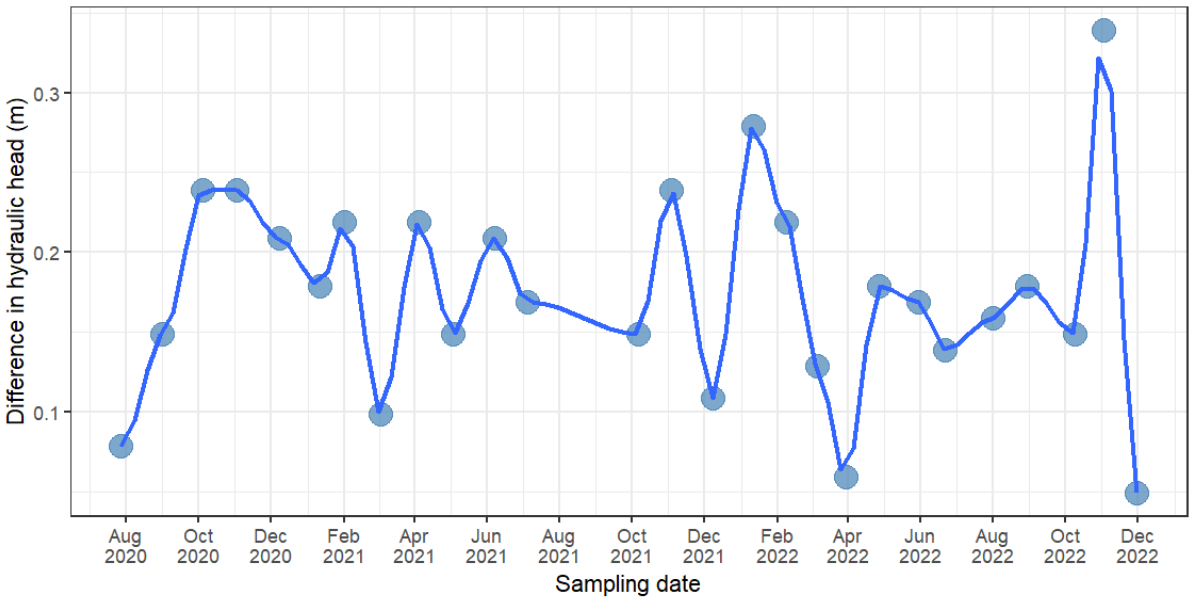

Figure S2: Differences in hydraulic head values between groundwater monitoring site D05 closest to the surface water ESW and groundwater site D15, for period summer 2020-winter 2022.

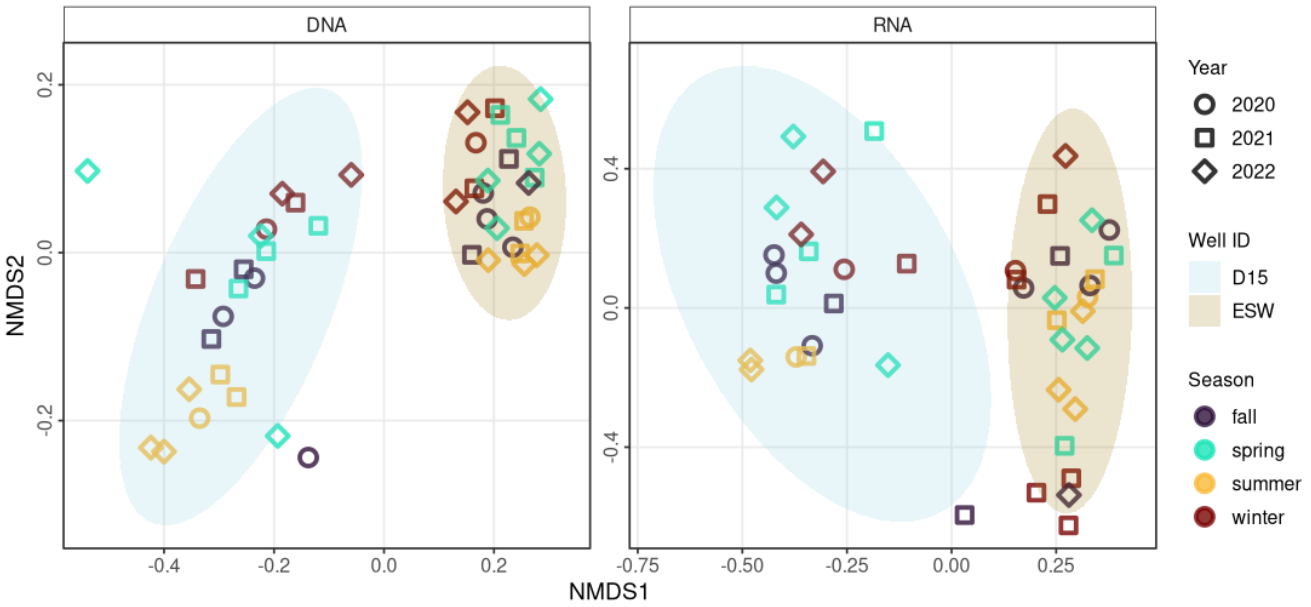

Figure S3: NMDs of Jaccard distances for both molecular methods, according to sampling site, season and year.

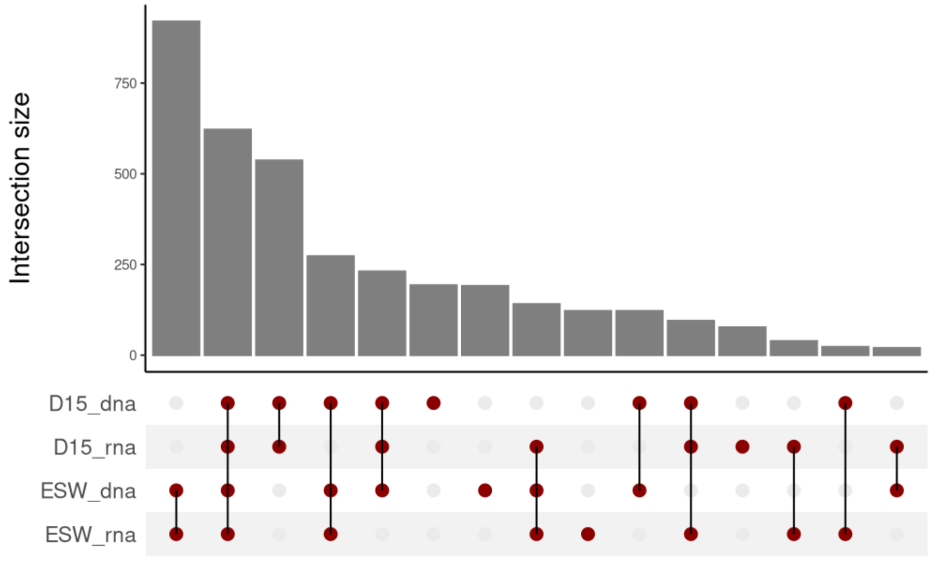

Figure S4: Shared protistan ASVs across the two sampling sites and two molecular methods. The bar plot on the top shows the size of ASV intersection across group combinations, the bottom indicates the groups involved in each intersection (dark red dots connected by lines).

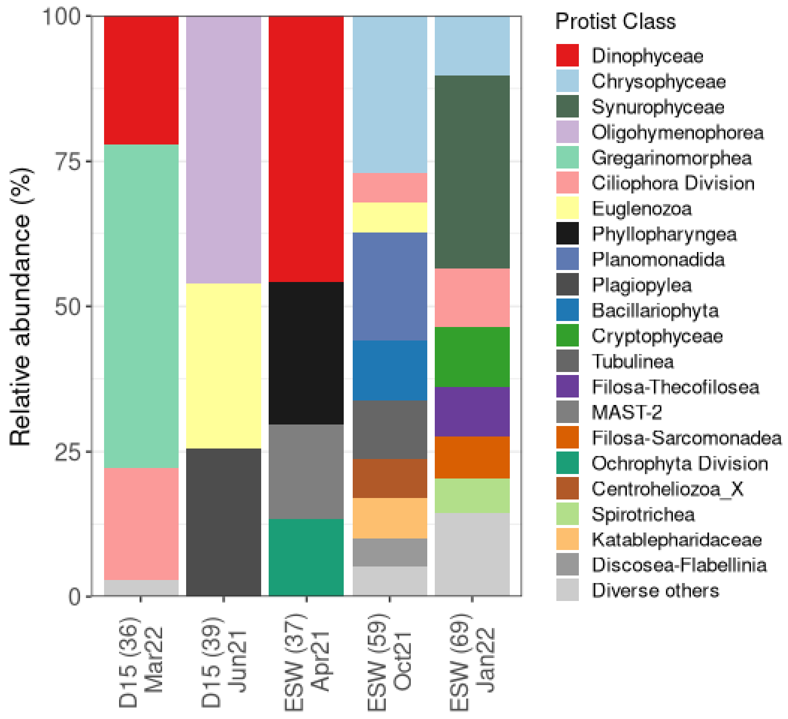

Figure S5: Relative abundances of classes identified in sampling points with <100 sequence counts. Sampling site and date of sampling together with read counts in paretheses are written on x-axis.

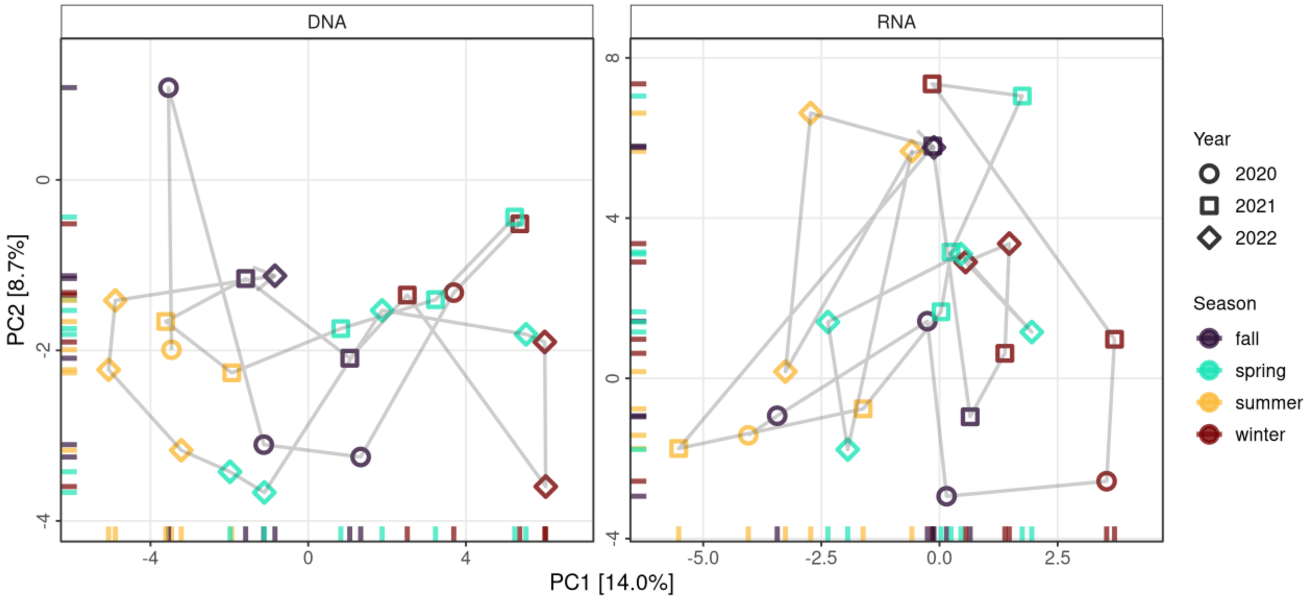

Figure S6: PCA of each sampling site's combined protist compositions of surface water and groundwater for both molecular methods used, according to the season and year. The sampling times are represented as a sequential line between the points.
